# Vagal Nerve Stimulation Therapy: What Is Being Stimulated?

**DOI:** 10.1371/journal.pone.0114498

**Published:** 2014-12-05

**Authors:** Guy Kember, Jeffrey L. Ardell, John A. Armour, Mair Zamir

**Affiliations:** 1 Department of Engineering Mathematics, Dalhousie University, Halifax, Nova Scotia, Canada; 2 Department of Biomedical Sciences, East Tennessee State University's Quillen College of Medicine, Johnson City, Tennessee, United States of America; 3 Department of Applied Mathematics, Western University, London, Canada; 4 Department of Medical Biophysics, Western University, London, Canada; The University of Queensland, Australia

## Abstract

Vagal nerve stimulation in cardiac therapy involves delivering electrical current to the vagal sympathetic complex in patients experiencing heart failure. The therapy has shown promise but the mechanisms by which any benefit accrues is not understood. In this paper we model the response to increased levels of stimulation of individual components of the vagal sympathetic complex as a differential activation of each component in the control of heart rate. The model provides insight beyond what is available in the animal experiment in as much as allowing the simultaneous assessment of neuronal activity throughout the cardiac neural axis. The results indicate that there is sensitivity of the neural network to low level subthreshold stimulation. This leads us to propose that the chronic effects of vagal nerve stimulation therapy lie within the indirect pathways that target intrinsic cardiac local circuit neurons because they have the capacity for plasticity.

## Introduction

Vagal nerve stimulation is currently being used to treat epilepsy and is being explored for the treatment of heart disease and other ailments [1]. Specifically, it is being utilized to treat patients experiencing cardiac arrhythmias [2], [3] and heart failure [4], [5]. This procedure involves delivering electrical stimuli to the cervical vagal sympathetic complex (VSC) [6], [7]. VNS therapy, which involves delivering electrical current to the VSC, has been shown to impart some benefit to cardiac arrhythmia and heart failure patients [3], [4], [8], [9]. The mechanisms by which this benefit is obtained are not fully understood [3], [8], [10], [11]. Furthermore, the level and mode of current delivery required to obtain optimum clinical outcome is not known [4].

The VSC contains axons that carry cardiovascular sensory feedback to the medulla along with both parasympathetic and sympathetic motor axons that project to neurons on the heart [12]. These parasympathetic preganglionic efferent axons synapse with (1) intrinsic cardiac parasympathetic efferent postganglionic neurons that innervate the cardiac musculature and (2) intrinsic cardiac local circuit neurons [13]. The second are much more numerous than the first [13]. For the purpose of the present paper we shall refer to the first of these as a “direct” parasympathetic pathway because its axons synapse directly with cardiac cholinergic postganglionic neurons. The second shall be referred to as an “indirect” parasympathetic pathway because its axons synapse with intrinsic cardiac local circuit neurons that in turn modulate cardiac motor neurons. The main feature of the direct pathway is that it involves single pre-to-postganglionic synapses, while the indirect pathway involves multiple synapses organized as a neural network.

When the complex structure of the VSC is stimulated, all of its elements may be affected. Afferent axons along with efferent cholinergic and adrenergic axons may become activated. As a consequence, the corresponding alteration in cardiac indices elicited in the process are multifaceted. These processes are at present difficult to resolve in the intact state. Thus the questions of what is being affected by vagal stimulation therapy and how to interpret the resulting changes in cardiac indices, particularly heart rate, remain unresolved. The prospects for resolving these questions in an experiment are clearly limited. Added to these difficulties is the fact that the neural control system of the heart is a multilevel network [14]. As such, it is not known how effects of VNS therapy influence the dynamics of cardiac control. Similar questions arise in cranial nerve stimulation which is a growing therapeutical strategy for treating epilepsy and various psychiatric disorders [15]–[22].

In this paper we present a model in which the direct and the indirect pathways of VNS therapy on individual elements of the cardiac hierarchy can be isolated. This permits understanding the putative complexity of cardiac responsiveness to such therapy. By selectively activating the different elements of the VSC and observing their individual impact on the cardiac control hierarchy, our aim is to establish a relationship between stimulation of the various components of the VSC and the ensuing effects within the neural control hierarchy of the heart.

## Neural Control of the Heart

In the classical view, neural control of the heart was explained mainly in terms of central neural command, specifically in terms of medullary and spinal cord autonomic efferent preganglionic neurons targeting efferent postganglionic neurons that innervate the heart [23]. In recent years it has become clear that there is a 3-level hierarchy of cardiac control, two of these residing outside the central nervous system, specifically (1) within the intrinsic cardiac nervous system and (2) within intrathoracic extracardiac ganglia. The results we present in this paper are based on a model of this 3-level control system which has previously been shown to explain heart rate phenomena which could not be explained with the classical view of cardiac control by central command [14], [24], [25].

### Neural Network

*A key feature of this model is that each of the three levels of control is assumed to consist of a population of neurons which influence and are influenced by each other at their own level of control as well as at adjacent levels. We refer to this collectively as the “neural network”.*

The network we use in this paper has 3*N* neurons equally divided among three levels of control which we shall refer to as levels 1,2,3, “bottom”, “middle”, “top”, or “cardiac”, “intrathoracic”, “central”, respectively. Two indices, 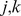 are used to identify the 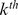 neuron at the 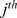 level. The state of activity (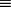 level of discharge) of neuron 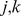 at time interval 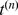 is denoted by 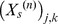 for sympathetic control, 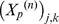 for indirect parasympathetic control, and 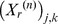 for direct parasympathetic control as explained in more detail below. All neural activity is scaled to range between 1.0 when a neuron is most active and 0.0 when it is inactive.

### Heart Rate Control Algorithm

*Broadly speaking, the neural network receives continuous neuronal updates of current demand for blood flow and current heart rate, and processes this state of the system at each time interval to produce an appropriate change in heart rate. The main result of this process, which is a key feature of the model, is that demand for blood flow does not proceed directly to the heart or to central command but to the neural network as a whole. The way this occurs is described briefly below, more details can be found in [14], [24].*

Heart rate is constrained to lie between a prescribed base value and a prescribed maximum value. A scaled heart rate *H* is used such that *H* = 1.0 when heart rate is maximum and *H* = 0.0 when heart rate is at base value.

The dynamics of the neural network unfold piecewise at consecutive time intervals 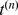, 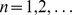, where demand for blood flow and current heart rate are used as inputs and an incremental “move” 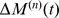 is produced as output.

The average activity of sympathetic efferent postganglionic neurons at the cardiac level is used as an external input to motor neurons at the cardiac level such that the efferent sympathetic neural input 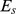 to the heart satisfies
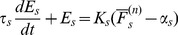
(1)where 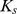 is a sympathetic gain, 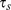 is a sympathetic time constant, 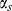 is a sympathetic reference level and 
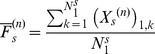
(2)where 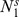 is the number of sympathetic neurons at the cardiac level.

Indirect parasympathetic efferent preganglionic neural activity 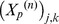 passes through the neural network and appears as input to parasympathetic efferent postganglionic motor neurons at the cardiac level such that the efferent indirect parasympathetic neural input 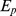 to the heart satisfies 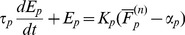
(3)where 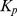 is an indirect parasympathetic gain, 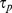 is indirect parasympathetic time constant, 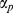 is the indirect parasympathetic reference level and 
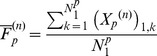
(4)where 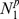 is the number of indirect parasympathetic neurons at the cardiac level.

Direct parasympathetic activity 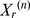 bypasses the neural network and proceeds directly from central command to the heart such that efferent direct parasympathetic neural input 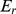 to the heart satisfies 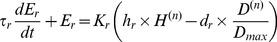
(5)where 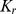 is a direct parasympathetic gain, 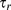 is direct parasympathetic time constant, 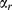 is direct parasympathetic reference level, 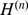 and 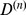 are prevailing heart rate and blood demand at time interval 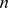 and 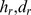 are “sensitivities” of direct parasympathetic neurons to heart rate and blood demand, respectively.

Finally, the change in heart rate or “move” 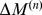 at time interval 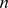 is the net of the above three efferent contributions 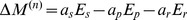
(6)where 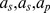 are constants.

*Within* each time interval 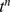, heart rate is a continuous function of time governed by a first order linear system 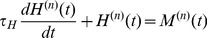
(7)where 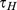 is a time constant and 
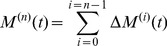
(8)


For simplicity, this time dependence will not be shown explicitly in what follows but will be implicit within each time interval 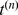.

### Neural Discharge

*In general, the level of activity of a neuron (neural discharge) is determined by the demand for blood flow but is also affected by current heart rate and by the level of activity of neighboring neurons.*

Specifically, the action potential generated by a neuron 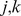 within the network in time interval 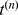 is represented by the state of activity 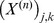 of that neuron. A change in the state of activity of the neuron due to these effects shall be denoted respectively by 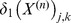, 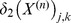, 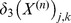, and total change by 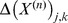.

*We distinguish between two types of neurons [26], [27]: “heart-rate neurons” which are affected by only current heart rate and the activity of neighboring neurons, and “blood-demand neurons” which are affected by only demand for blood flow and the activity of neighboring neurons.*

Thus the change in the state of activity of a sympathetic neuron 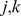 is given by
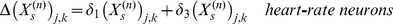
(9)

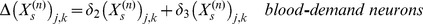
(10)and the corresponding change in the state of activity of an indirect parasympathetic neuron is similarly given by 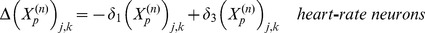
(11)

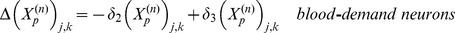
(12)


The extent to which heart rate *H* and blood demand *D* affect the state of activity of a sympathetic or parasympathetic efferent neuron 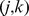 depend on heart rate and blood demand “sensitivities” 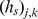 and 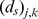 such that for a sympathetic neuron 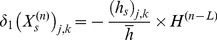
(13)

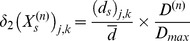
(14)and for a parasympathetic neuron 
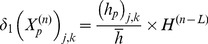
(15)

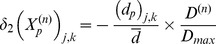
(16)where 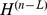 is heart rate delayed by a time constant 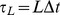 and 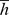 is the mean of 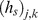 and 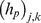 over the entire network.

*The way in which the state of activity of a neuron 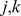 is influenced by the states of activity of its neighboring neurons is what we have referred to as “networking”. It is a key feature of the model whereby every neuron within the network influences and is influenced by other neurons.*

Networking is represented by 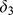 in Eqs.9,10,11,12. The extent of networking between a particular neuron 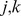 and its 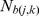 neighboring neurons 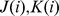, 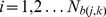 (upper case 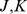 being used to represent *neighboring* neurons) is determined by the weighted sum of the difference prevailing in time interval 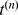 between the state of activity of that neuron and the states of activity of the neighboring neurons. Thus, for a sympathetic neuron we have 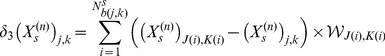
(17)and for a parasympathetic neuron 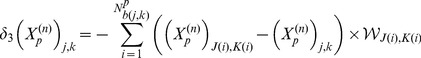
(18)where 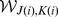 is a weighting, a measure of the connectivity between neuron 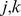 and its neighbor 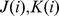.

## Animal Experiments

Vagal stimulation experiments described below were performed on intact dogs specifically to demonstrate threshold phenomena in observed heart rate changes as the level of VNS stimulation was gradually increased from zero. The individual animals (n = 8) were mongrel adults weighing between 20–25 kg. Response to VNS was examined in the conscious and anesthetized states. We have found in previous studies that both in the conscious (see Experimental Results section) and anesthetized [12], [28] states the system maintains bidirectional sensitivity. The main results we present are based on the response to VNS from an animal that was anesthetized. We also show the average response to VNS from 7 animals that were in the conscious state. Under aseptic surgical conditions and isoflurane (2%) anesthesia, the latter group received VNS therapy system implant (Demipulse 103 implantable stimulator with Model 304 bipolar helical cuff; Cyberonics, Houston, TX) involving the right cervical vagus nerve. Following a two week recovery period, animals were trained to the Pavlov stand. Following an initial 4 week titration period, VNS response curves were determined at 10 Hz, 500 µsec pulse width, with a 18% duty cycle (14 sec on, 66 sec off) and with current randomized between 0.25 to 3.50 mA. Heart rate responses were quantified by the percent change from the baseline in response to VNS as shown in the results.

All experiments were performed in accordance with the guidelines for animal experimentation described in the “Guide for the Care and Use of Laboratory Animals: Eighth Edition, 2010”. The Institutional Animal Care and Use Committee of East Tennessee State University approved these experiments.

### Instrumentation

Animals were pre-medicated with sodium thiopental (15 mg/kg, i.v), intubated and anesthetized using 2% isoflurane. The left femoral vein was catheterized to allow fluid replacement as well as the administration of anesthetic and pharmacological agents. Left ventricular chamber pressure was measured via a 5-Fr Mikro-Tip pressure transducer catheter (Millar Instruments, Houston, TX) inserted into that chamber via the left femoral artery. The right femoral artery was catheterized to monitor aortic pressure using another Mikro-Tip transducer. Heart rate was monitored via ECG lead II. All hemodynamic data were digitized (Cambridge Electronic Design power 1401 acquisition system with Spike 2 software) for subsequent off-line analysis.

Following a ventral midline incision, both cervical vagosympathetic nerve trunks were isolated. For the right cervical vagosympathetic trunk, a bipolar helical cuff stimulation electrode (Cyberonics, Inc) was placed around that nerve, with the distal electrode positioned distal to the head. The lead was secured in place and connected to a Grass S88 stimulator via a Grass PSIU6 current isolation unit.

Throughout all surgical procedures, depth of anesthesia was assessed by monitoring corneal reflexes, jaw tone and alterations in cardiovascular indices. Body temperature was monitored rectally and maintained steady via a circulating water heating pad (T/Pump, Gaymar Industries Inc., Orchard Park, NY). Respiration was controlled using an artificial ventilator (at 12–16 cycles/min) supplied with oxygen. Acid-base status was evaluated hourly (Irma TruePoint blood gas analyzer, International Technidyne Corp., Edison NJ); tidal volume was adjusted and bicarbonate infused as necessary to maintain blood gas homeostasis. Following completion of the surgery, anesthesia was changed to alpha-chloralose (75 mg/kg i.v. bolus), with continuous infusion (16 mg/kg/hr) adjusted as required throughout the duration of the study.

### Experimental Protocol

The right cervical vagus was stimulated electrically with current intensities ranging from 0.25 mA to 3.5 mA in increments of 0.25 mA. We employed a stimulus isolation unit (Grass model PSIU6 photoelectric isolation unit) which was connected to the Grass stimulator to active the vagosympathetic complex with constant current for anesthetized studies. For conscious animals, we used a Cyberonics Demipulse 103 implantable stimulator to deliver VNS. Each 122 *s* stimulation cycle consisted of 500 *µs* duration pulses delivered at a frequency of 10 Hz over a period of 14 s (VNS on) followed by a period of 108 s of zero stimulation (VNS off). The intensity of electric current used in each of these 122 s stimulation cycles of VNS on/off was chosen randomly within the range 0.25 mA to 3.5 mA in increments of 0.25 mA. Digitized hemodynamic data for off-line analysis consisted of beat-to-beat heart rate, blood pressure, left ventricular pressure and its time derivative dp/dt.

## Results

### Experimental Results

**Figure 1:**
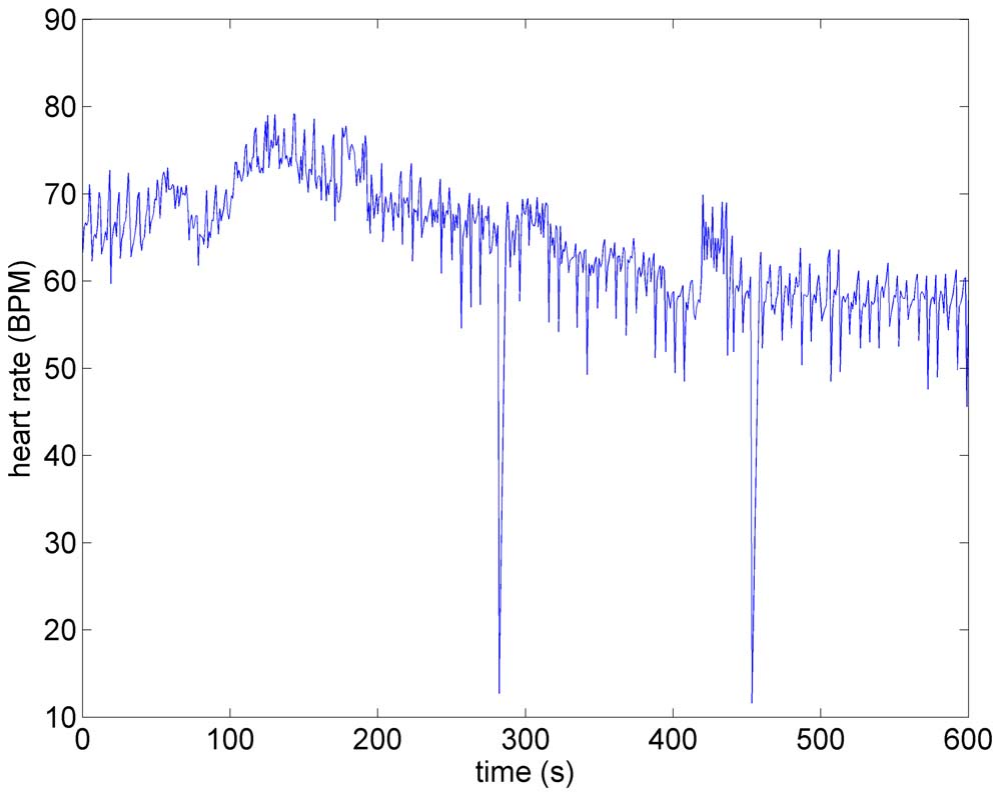
Heart rate in an anesthetized canine under baseline conditions, in the absence of VNS. Note the variations in heart rate that occur in the normal state.

**Figure 2:**
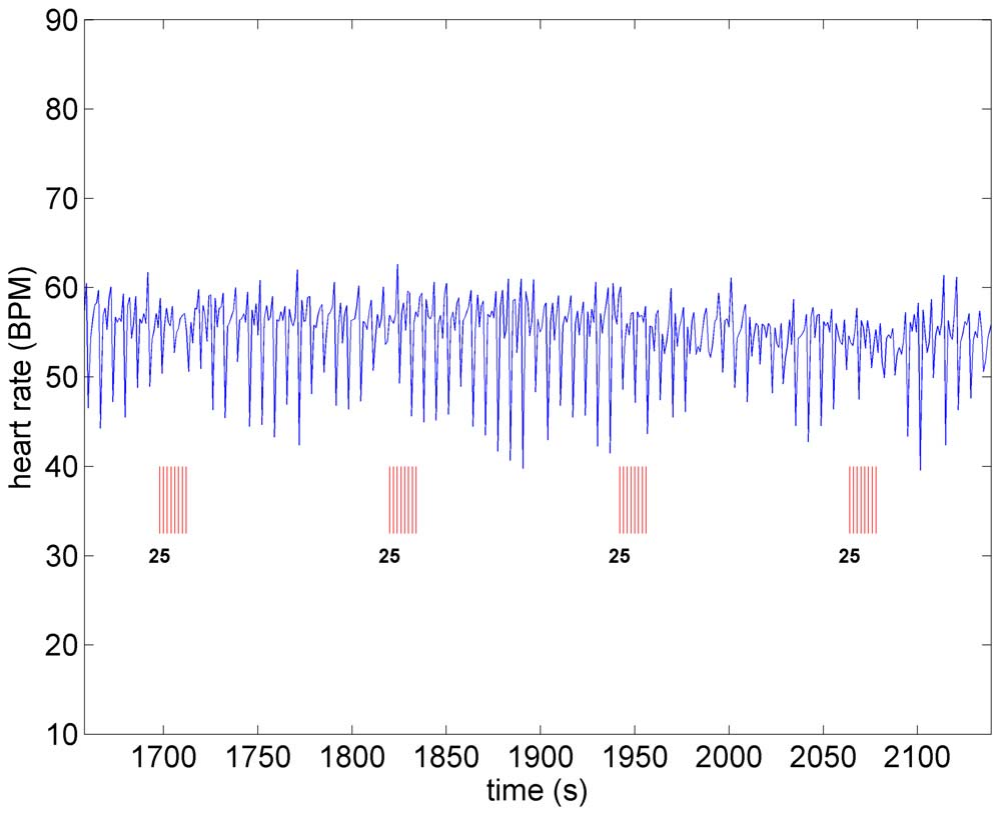
Heart rate under low level stimulation (0.25 *mA*) in the same animal as in Figure 1. No discernible heart rate changes are observed.

**Figure 3:**
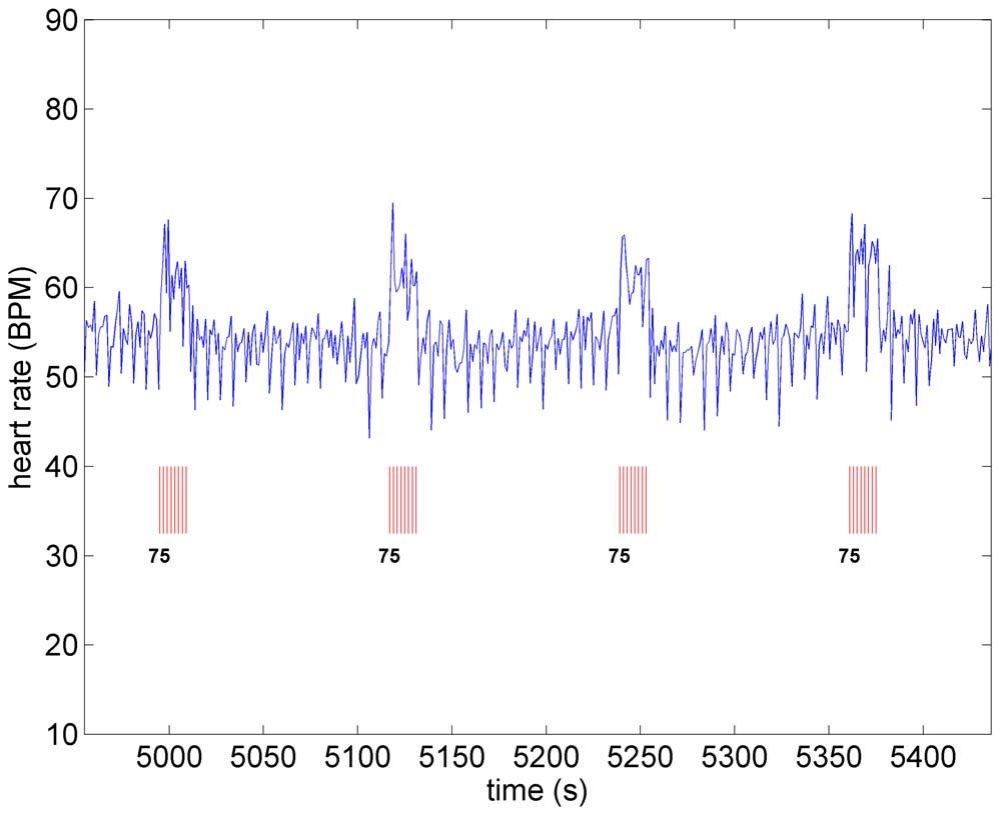
Heart rate under moderate level stimulation (0.75 *mA*) in the same animal as in Figure 1. Pronounced tachycardia is observed.

**Figure 4:**
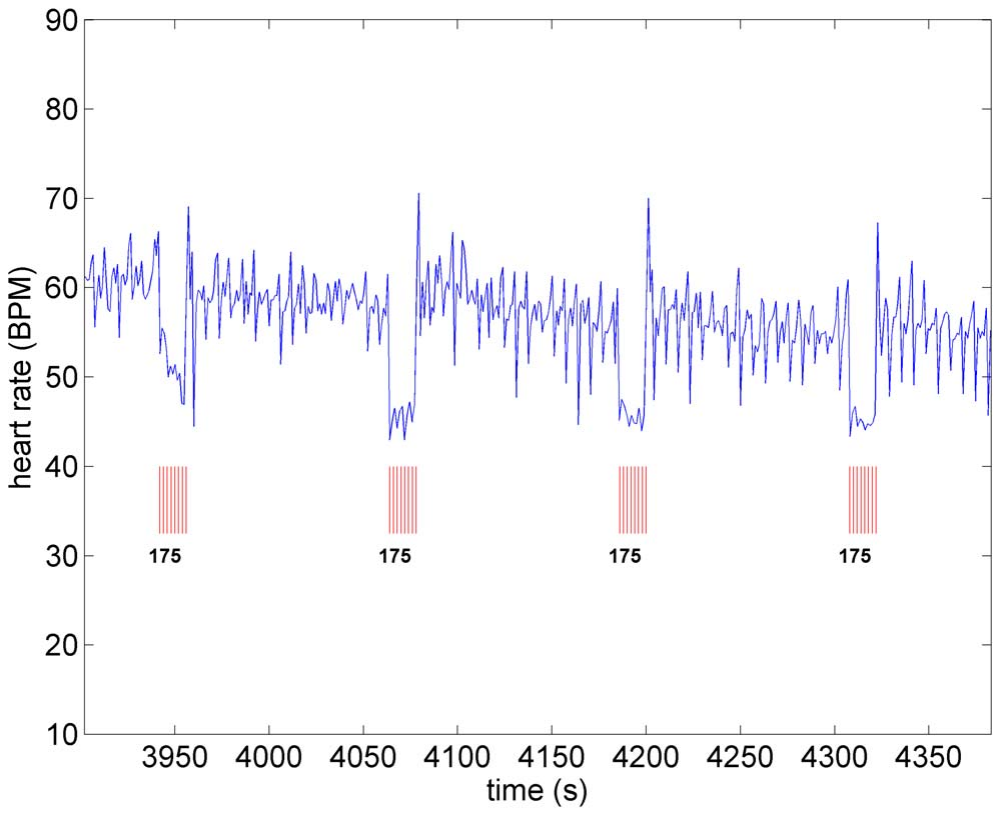
Heart rate as the level of stimulation is increased from that in Figure 3 (from 0.75 *mA* to 1.75 *mA*). Pronounced bradycardia is observed.

**Figure 5:**
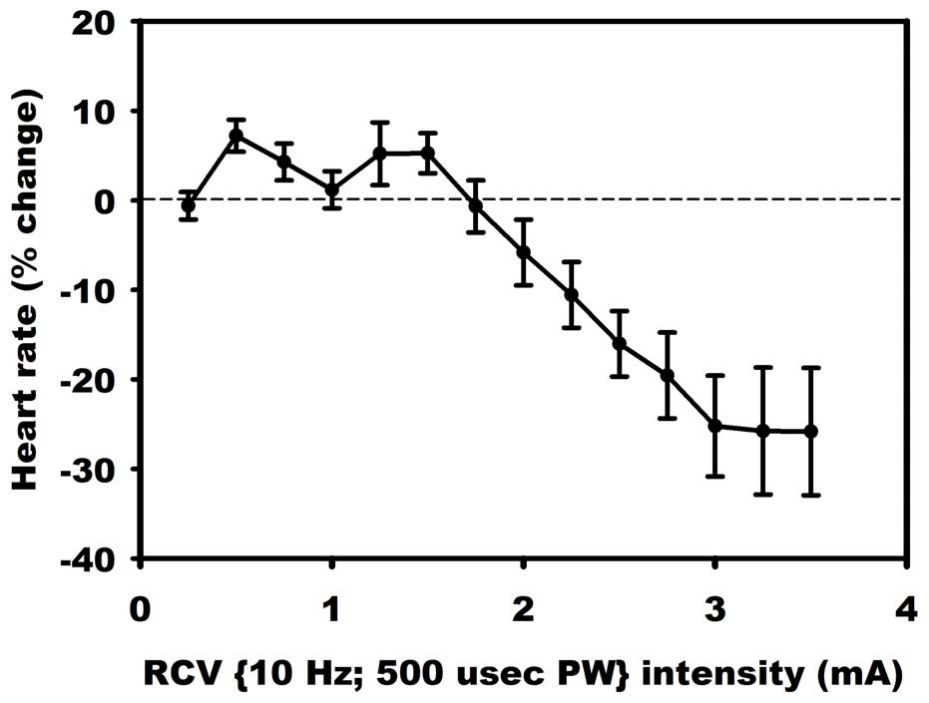
Average response to VNS from 7 animals that were in the conscious state.

A key feature of VNS is that the response of heart rate to stimulation is not a “predictable” function of the intensity of stimulation. This is illustrated in Figures 1–4 in which the intensity of stimulation was at baseline (0.0 mA) followed by 0.25 mA, 0.75 mA, and 1.75 mA, respectively. At the lowest intensity, heart rate response to stimulation is inconsistent and barely noticeable. At the intermediate intensity of 0.75 mA there is a clear and consistent tachycardia in response to stimulation, while at 1.75 mA there is a clear and consistent bradycardia. While these results were based on an anesthetized animal, the average response to VNS from 7 animals that were in the conscious state is shown in Figure 5.

### Model Simulation Results

The model simulations described in what follows were designed to examine the interplay between the direct and the indirect pathways to the heart as the VSC is stimulated at different intensities. While in the experiment these pathways cannot be separated, in the model they can be activated with different intensities and independently from each other or can actually be turned on or off entirely.

Specifically, “stimulation” of the *indirect* component is implemented by a decrease in the sensitivities of heart rate neurons to current heart rate, and an increase in the sensitivities of blood demand neurons to current blood demand. The two effects are applied simultaneously and continuously while stimulation is ON, and to both sympathetic and parasympathetic local circuit neurons. Stimulation of the *direct* component is implemented by an increase in the intensity of the efferent direct parasympathetic input to the heart (*a_r_* in Eq.6).

*Baseline:* The amounts by which the sensitivities to current heart rate and current blood demand are changed, as described above, determine the “intensity of stimulation” in the model simulations to be described in what follows. It is important to point out that while the progression of this stimulation intensity in the model from zero to higher levels will mimic the corresponding progression of stimulation in the experiment, it is not possible to actually relate the levels of these two stimulation intensities in any direct way. Instead, in what follows we present the effect on heart rate only as the stimulation intensity is progressively increased from zero.

**Figure 6:**
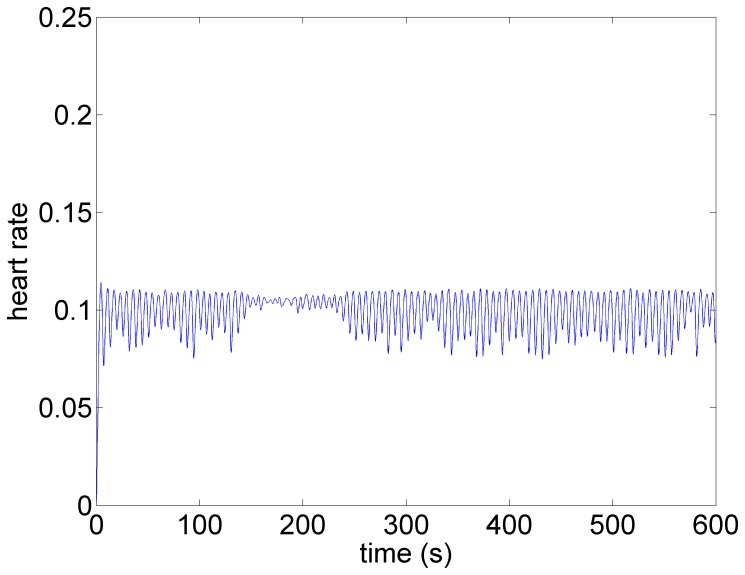
Model simulation under baseline conditions (zero stimulation). The oscillatory pattern and the variability in that pattern is similar to that observed in the experiment (Figure 1) and is typical at low blood demand and in the presence of low level noise within the system [14]. Brief intervals of resonance, whereby the oscillations are subdued, can be observed in both cases.

The pattern of heart rate with zero stimulation is shown in Figure 6. The oscillatory pattern and the variability in that pattern is similar to that observed in the experiment (Figure 1) and is typical at low blood demand and in the presence of low level noise within the system [14]. Brief intervals of resonance whereby the oscillations are subdued can be observed in both cases.

**Figure 7:**
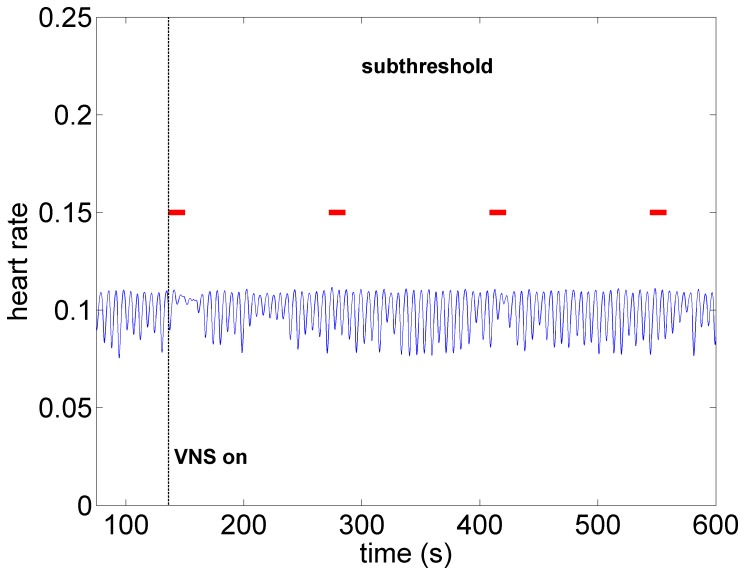
Model simulation under subthreshold conditions whereby the direct component of the VSC is not being activated but the indirect component and therefore the local circuit elements of the neural network are being activated at low intensity. Here, as in the experiment (Figure 2), there are no discernible changes in heart rate. Red bars indicate time intervals when VNS is on.

*Subthreshold:* As the level of stimulation is increased from zero in the model the effect on heart rate is barely visible, as observed in Figure 7. Here the direct component of the VSC is turned off and the indirect component is minimally stimulated as described above. We refer to this set of conditions as “subthreshold” in the sense that no qualitative changes in heart rate are discernible at this level of stimulation. It is important to point out that while the direct component of the VSC is not being activated at this level of stimulation, the indirect component of the VSC and therefore the local circuit elements of the neural network are being activated. This has important implications which will be discussed later.

**Figure 8:**
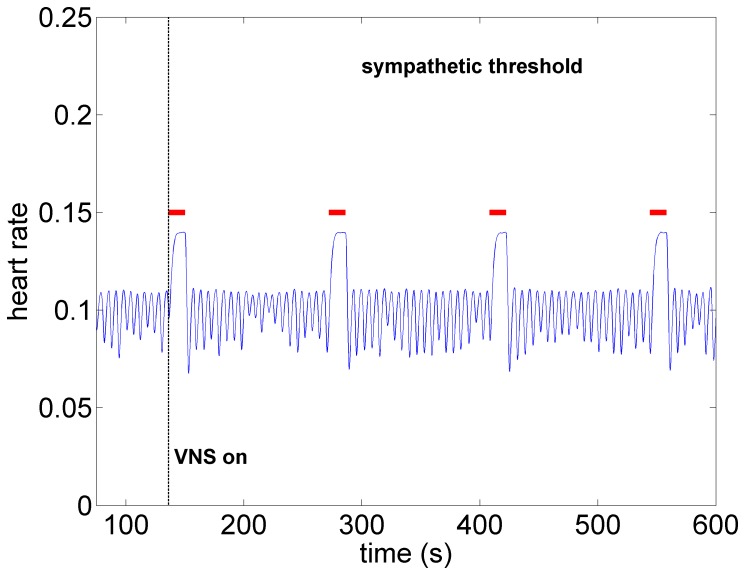
Model simulation under sympathetic threshold conditions whereby the direct component of the VSC is not being activated but the indirect component is being activated at higher intensity than that in Figure 7. Pronounced tachycardia is observed, similar to that seen in the experiment under moderate intensity stimulation (Figure 3). Red bars indicate time intervals when VNS is on.

*Sympathetic Threshold:* As the level of stimulation is increased above the subthreshold level, but with the direct component of the VSC remaining OFF, a significant tachycardia occurs as shown in Figure 8. Within the construct of the model, this is clearly because we have chosen to make the indirect component of the VSC dominant over the direct element. For this reason we refer to this choice of relative influence of the two pathways as the “sympathetic threshold”, as indicated in the figure. It is important to note that the term “sympathetic threshold” is here not intended to imply that the sympathetic network is being directly stimulated at this point but rather that the response to the twin effects of a reduced feedback to heart rate neurons at the cardiac level and increased demand to blood demand neurons at the central level is being dominated by the sympathetic network.

**Figure 9:**
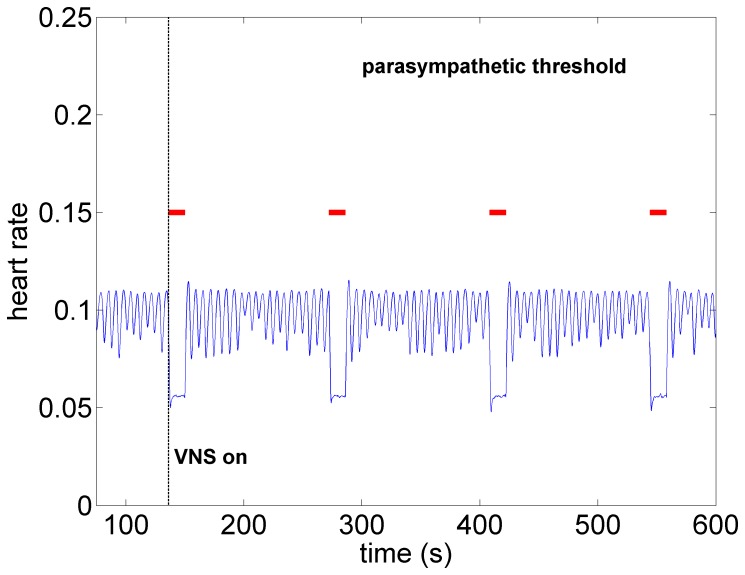
Model simulation under parasympathetic threshold conditions whereby the direct component of the VSC is now being activated while the indirect component being maintained at the same activation intensity as in Figure 8. Pronounced bradycardia is observed, similar to that seen in the experiment under high intensity stimulation (Figure 4). Red bars indicate time intervals when VNS is on.

*Parasympathetic Threshold:* Finally, as the level of activation of the *direct* component of the VSC is gradually increased from zero, with the level of stimulation of the indirect component being maintained under the sympathetic threshold conditions, the parasympathetic elements of the direct component of the VSC become dominant. The result is a significant bradycardia as shown in Figure 9, hence we refer to this as the “parasympathetic threshold”, again, implying only that the *response* to stimulation is now being dominated by the direct component of the VSC.

## Discussion and Conclusions

The clinical efficacy of vagal nerve stimulation for the control of heart rate is not understood at present because of the following major issues. First, it is not known precisely which components of the VSC are being activated when the stimulation is applied in the clinical setting. Second, it is not known how the effects of stimulation differentially influence the three levels of neural control hierarchy of the heart. Third, only changes in heart rate at the time of intermittent stimulation are currently available as a guide as to the efficacy of the procedure. Fourth, the long term effects of stimulation on the heart are not known. Indeed, the basis or mechanism for such long term effects have not been established.

In the face of these issues we propose a mathematical model of the neural control hierarchy of the heart in which neural pathways under different scenarios of vagal nerve stimulation can be separated and identified. The motivation for this approach is that the above issues are difficult to resolve experimentally because the components of the VSC are not accessible individually. Thus the model not only provides this access but allows individual activation of the different components of the VSC.

At the core of our findings, based on the mathematical model, is a distinction that must be made between *direct* parasympathetic pathways whereby cardiac motor postganglionic neurons are targeted directly, and *indirect* sympathetic and parasympathetic pathways which interact indirectly with a population of local circuit neurons on the heart.

The application of VNS in the model is implemented differently for the direct and indirect pathways. For the direct pathway, stimulation is implemented by simply increasing the intensity of activation. By contrast, stimulation of the indirect pathway is implemented by (i) a reduction in sensitivity of heart rate neurons and (ii) an increase in sensitivity of blood demand neurons. In addition, in the model, heart rate neurons are located mainly at the level of the intrinsic nervous system while blood demand neurons are located mainly at the level of the central nervous system. Thus, VSC stimulation of the indirect pathway in the model is represented as a complex combination of a reduction in afferent feedback and an increase in efferent drive caused by an increase in central sensitivity to blood demand.

The model results indicate that at very low levels of stimulation, if only the indirect pathways through the VSC are activated, no discernible change in heart rate is produced. As the level of stimulation is gradually increased beyond a certain threshold, a clear tachycardia is observed. Then, as the intensity of the direct pathway is gradually increased, while the level of activation of the indirect pathways are maintained, another threshold emerges where the net effect on heart rate is reversed and a clear bradycardia becomes evident.

We have thus modeled the response to increased levels of activation of the VSC as the differential activation of different components of the VSC. To the extent that the model results exhibit features similar to those observed in the anesthetized animal, we are led to conclude that different components of the VSC may respond differentially to different levels of stimulation.

In particular, our interpretation of the bradycardia observed at higher levels of stimulation in the experiment is that the effect of stimulation is now dominated by the direct parasympathetic elements of the VSC whose intensity has been increased. On the other hand, our interpretation of the tachycardia observed at lower levels of stimulation in the experiment is that now the indirect elements of the VSC are primarily active. As stated earlier, in this case VNS stimulation in the model is represented as a complex combination of a reduction in afferent feedback and an increase in efferent drive caused by an increase in central sensitivity to blood demand. The net result is the observed increased heart rate.

**Figure 10:**
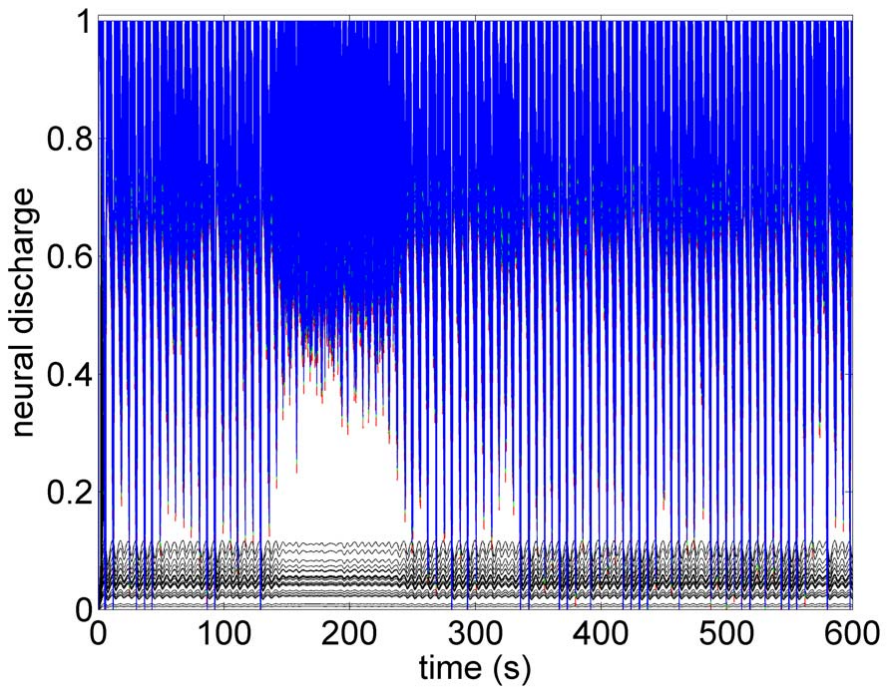
A map of neural activity (discharge) at the three levels of the neural network under the model baseline conditions. The range of activity is normalized to lie between 0 (no activity) and 1.0 (maximal activity). There are 600 neurons at each level of the network (Sympathetic neurons: blue  =  central, green  =  intrathoracic, red  =  cardiac. Black represents 100 parasympathetic neurons at the cardiac level.) The pattern of activity is consistent with the pattern of heart rate variability observed in Figure 6.

**Figure 11:**
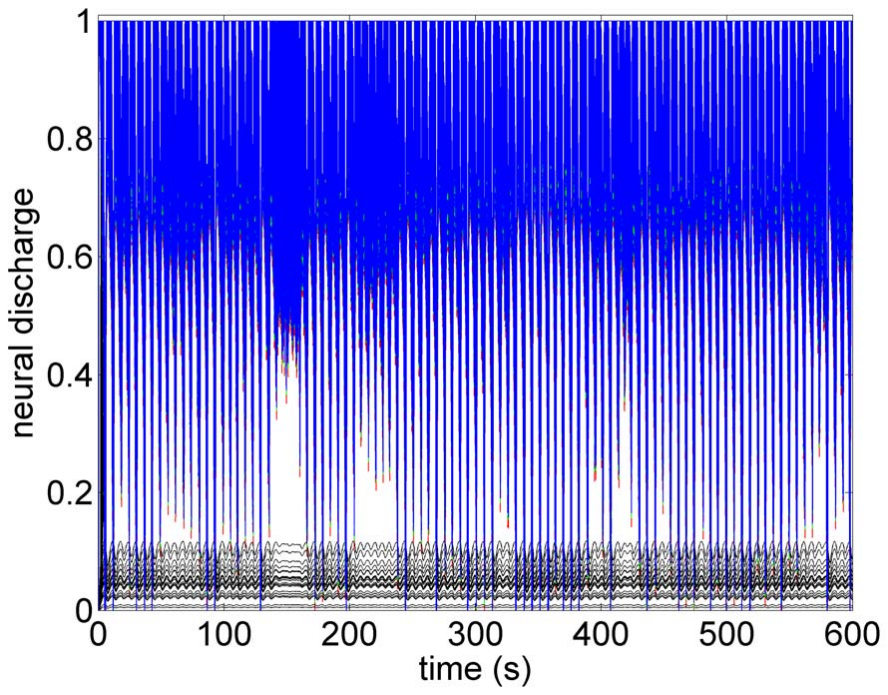
A map of neural activity (discharge) at the three levels of the neural network under the model subthreshold conditions and corresponding to the pattern of heart rate observed in Figure 7. Legend as in the caption of Figure 10.

**Figure 12:**
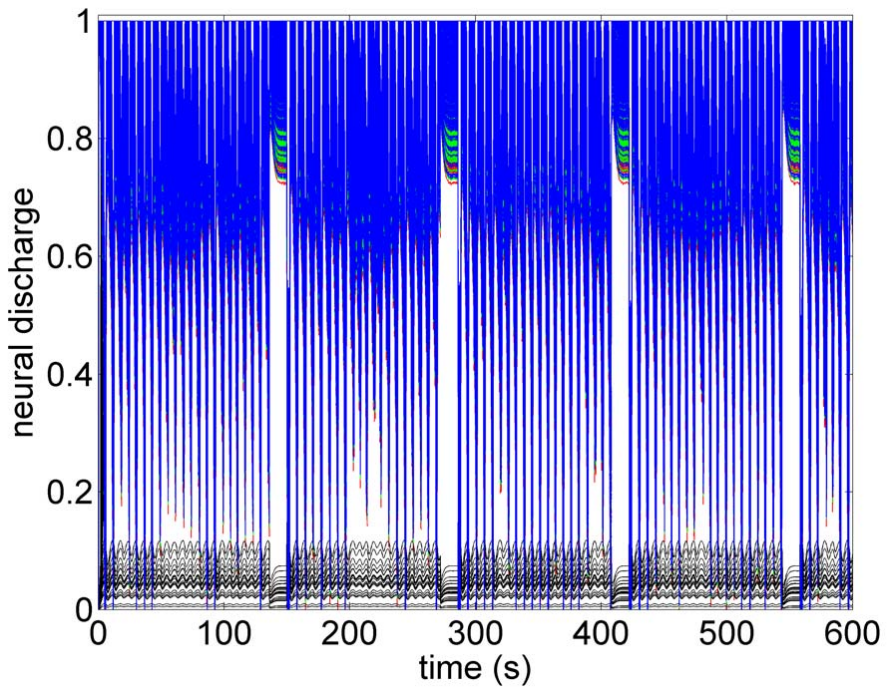
A map of neural activity (discharge) at the three levels of the neural network under the model sympathetic threshold conditions and corresponding to the pattern of heart rate observed in Figure 8. Tachycardia observed in Figure 8 is here seen to be the result of higher activity of sympathetic neurons at all three levels of the neural network, coupled with some suppression of parasympathetic activity induced mainly by withdrawal of afferent feedback at the cardiac level. Legend as in the caption of Figure 10.

**Figure 13:**
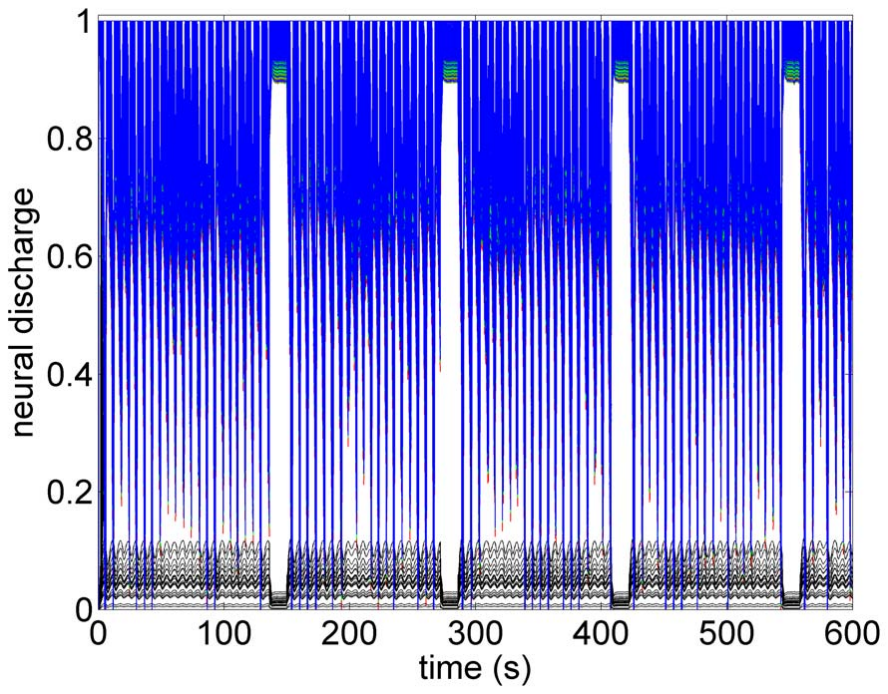
A map of neural activity (discharge) at the three levels of the neural network under the model parasympathetic threshold conditions and corresponding to the pattern of heart rate observed in Figure 9. Here the *direct* component of the VSC is stimulated, with the level of stimulation of the indirect component being maintained the same as under the sympathetic threshold conditions. The direct component of the VSC dominates, leading to the bradycardia observed in Figure 9. Legend as in the caption of Figure 10.

**Figure 14:**
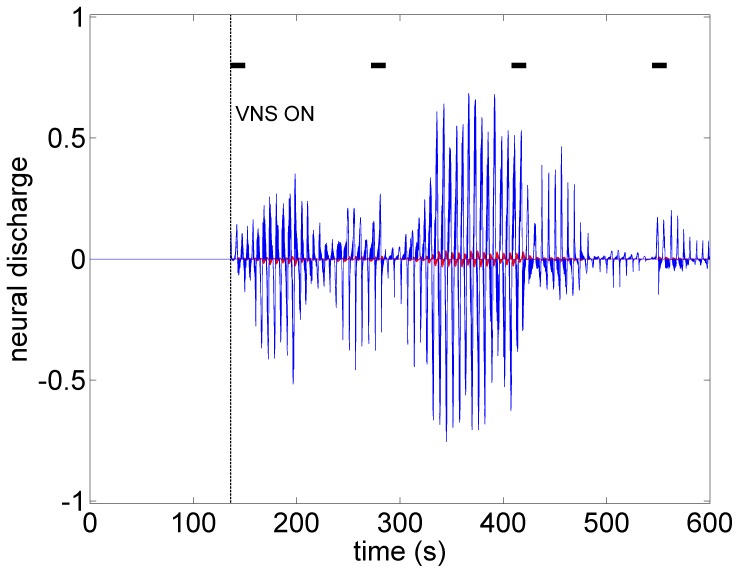
The difference between neural activity under baseline and subthreshold conditions, obtained by subtracting the map in Figure 6 from that in Figure 7. The figure demonstrates that while there were no discernible effects of subthreshold stimulation on either heart rate or neural discharge, there was considerable difference in neural activity under subthreshold conditions compared with those at baseline. In this figure the activities of all sympathetic neurons are shown in blue and the parasympathetic in red. Black bars indicate time intervals when VNS is on.

What the mathematical model also provides beyond what is available in the animal experiment is the simultaneous assessment of neuronal activity throughout the entire cardiac neuronal hierarchy. This is illustrated in Figures 10–13 which correspond to the baseline and threshold conditions seen in Figures 6–9. These figures show clearly that under subthreshold conditions, while there are no discernible changes in heart rate (Figure 7), there is considerable neural activity at all levels of the neural network (Figure 11). That this neural activity is different from baseline, is confirmed in Figure 14 which shows the difference between the level of discharge of each neuron at baseline and at subthreshold conditions.

The sensitivity of the neural network to low level subthreshold stimulation leads us to propose that the chronic effects of VNS therapy lie within the *indirect* pathways because plasticity and the potential for remodeling reside within the local circuit neurons of the intrinsic cardiac system and within the neural network comprising the indirect pathways [24]. By inference, the prospects for long term effects of VNS therapy lie in low level, “subthreshold”, activation where the indirect “plastic” components of the control system are involved.

## References

[1] Groves DA, Brown VJ (2005) Vagal nerve stimulation: a review of its applications and potential mechanisms that mediate its clinical effects. Neuroscience and Biobehavioral Reviews 29:493–500. 15820552 10.1016/j.neubiorev.2005.01.004

[2] Ando M, Katare RG, Kakinuma Y, Zhang D, Yamasaki F, et al (2005) Efferent vagal nerve stimulation protects heart against ischemia-induced arrhythmias by preserving connexin43 protein. Circulation 112:164–170. 15998674 10.1161/CIRCULATIONAHA.104.525493

[3] Zhang Y, Mazgalev TN (2011) Arrhythmias and vagus nerve stimulation. Heart Fail Rev 16:147–161. 20559719 10.1007/s10741-010-9178-2

[4] De Ferrari GM, Crijns HJ, Borggrefe M, Milasinovic G, Smid J, et al for the CardioFit Multicenter Trial Investigators (2011) Chronic vagus nerve stimulation: a new and promising therapeutic approach for chronic heart failure. European Heart Journal 32:847–855. 21030409 10.1093/eurheartj/ehq391

[5] DiCarlo L, Libbus I, Amurthur B, KenKnight BH, Anand IS (2013) Autonomic regulation therapy for the improvement of left ventricular function and heart failure symptoms: the ANTHEM-HF study. J Card Fail 19:655–660. 24054343 10.1016/j.cardfail.2013.07.002

[6] Foley JO, DuBois F (1937) Quantitative studies of the vagus nerve in the cat: I. The ratio of sensory motor studies. J Comp Neurol 67:49–67.

[7] Bailey P, Bremer FA (1938) Sensory cortical representation of the vagus nerve. J Neurophysiol 1:405–412.

[8] Schwartz PJ, De Ferrari GM, Sanzo A, Landolina M, Rordorf R, et al (2008) Long term vagal stimulation in patients with advanced heart failure: first experience in man. Eur J Heart Fail 10:884–891. 18760668 10.1016/j.ejheart.2008.07.016

[9] Zhang Y, Popovic ZB, Bibevski S, Fakhry I, Sica DA, et al (2009) Chronic vagus nerve stimulation improves autonomic control and attenuates systemic inflammation and heart failure progression in a canine high-rate pacing model. Circ Heart Fail 2:692–699. 19919995 10.1161/CIRCHEARTFAILURE.109.873968

[10] Osman F, Kundu S, Tuan J, Jeilan M, Stafford PJ, et al (2010) Ganglionic plexus ablation during pulmonary vein isolationpredisposing to ventricular arrhythmias? Indian Pacing and Electrophysiol J 10:104–107. 20126597 PMC2811210

[11] Kasanuki H, Ohnishi S, Ohtuka M, Matsuda N, Nirei T, et al (1997) Idiopathic ventricular fibrillation induced with vagal activity in patients without obvious heart disease. Circulation 95:2277–2285. 9142005 10.1161/01.cir.95.9.2277

[12] Randall WC, Armour JA (1977) Gross and microscopic anatomy of the cardiac innervation. In: Neural Regulation of the Heart. Randall WCEditor, Oxford Univ Press, N.Y.

[13] Beaumont E, Salavatian S, Southerland EM, Vinet A, Jacquemet V, et al (2013) Network interactions within the canine intrinsic cardiac nervous system: Implications for reflex control of regional cardiac function. J Physiol 591:4515–4533. 23818689 10.1113/jphysiol.2013.259382 PMC3784196

[14] Kember G, Armour J, Zamir M (2011) Neural control of heart rate: the role of neuronal networking. J Theor Biol 277:41–47. 21354183 10.1016/j.jtbi.2011.02.013

[15] Cook IA, Espinoza R, Leuchter AF (2014) Neuromodulation for Depression: Invasive and Noninvasive (Deep Brain Stimulation, Transcranial Magnetic Stimulation, Trigeminal Nerve Stimulation). Neurosurgery Clinics of North America 25:103–116. 24262903 10.1016/j.nec.2013.10.002

[16] Howland RH (2014) Vagus Nerve Stimulation. Current Behavioral Neuroscience Reports 1:64–73. 24834378 10.1007/s40473-014-0010-5 PMC4017164

[17] Shiozawa P, Enokibara da Silva M, Cristina de Carvalho T, Cordeiro Q, Brunoni AR, et al (2014) Transcutaneous vagus and trigeminal nerve stimulation for neuropsychiatric disorders: a systematic review. Arquivos de Neuro-Psiquiatria 72:542–7. 25054988 10.1590/0004-282x20140061

[18] Zare M, Salehi M, Mahvari J, Najafi MR, Moradi A, et al (2014) Trigeminal nerve stimulation: A new way of treatment of refractory seizures. Advanced Biomedical Research 3:81. 24761389 10.4103/2277-9175.127994 PMC3988608

[19] Koo B, Ham SD, Sood S, Tarver B (2001) Human vagus nerve electrophysiologya guide to vagus nerve stimulation parameters. J Clin. Neurophysiol 18:429–433. 10.1097/00004691-200109000-00007 11709648

[20] Krahl SE, Senanayake SS, Handforth A (2001) Destruction of peripheral C-fibers does not alter subsequent vagus nerve stimulation induced seizure suppression in rats. Epilepsia 42:586–589. 11380564 10.1046/j.1528-1157.2001.09700.x

[21] Nahas Z, Marangell LB, Husain MM, Rush AJ, Sackeim HA, et al (2005) Two-year outcome of vagus nerve stimulation (VNS) for treatment of major depressive episodes. J Clin Psychiatry 66(9):1097–104. 16187765 10.4088/jcp.v66n0902

[22] Woodbury DM, Woodbury JW (1990) Effects of vagal stimulation on experimentally induced seizures in rats. Epilepsia 31 (Suppl 2)S7–S19. 2226368 10.1111/j.1528-1157.1990.tb05852.x

[23] Williamson JW, Fadel JP, Mitchell JH (2005) New insights into central cardiovascular control during exercise in humans: a central command update. Exp. Physiol 91:51–58. 10.1113/expphysiol.2005.032037 16239250

[24] Kember G, Armour J, Zamir M (2012) Dynamic neural networking as a basis for plasticity in the control of heart rate. J Theor Biol 317:39–46. 23041448 10.1016/j.jtbi.2012.09.024

[25] Kember G, Armour JA, Zamir M (2013) Neural control hierarchy of the heart has not evolved to deal with myocardial ischemia. Physiol. Genomics 45:638–644. 23695889 10.1152/physiolgenomics.00027.2013

[26] Turrigiano G, Nelson S (2004) Homeostatic plasticity in the developing nervous system. Nat Rev Neurosci 5:97–107. 14735113 10.1038/nrn1327

[27] Turrigiano G, Nelson S (2000) Hebb and homeostasis in neuronal plasticity. Curr Opin Neurbiol 10:358–364. 10.1016/s0959-4388(00)00091-x 10851171

[28] Randall WC, Ardell JL (1985) Selective parasympathectomy of automatic and conductile tissues of the canine heart. Am J Physiol 248:H61–H68. 3970176 10.1152/ajpheart.1985.248.1.H61

